# The effect of nonlinear pedagogy on the acquisition of game skills in a territorial game

**DOI:** 10.3389/fpsyg.2023.1077065

**Published:** 2023-02-06

**Authors:** Jia Yi Chow, Laurentius A. Meerhoff, Corliss Zhi Yi Choo, Chris Button, Benjamin Su-Jim Tan

**Affiliations:** ^1^Physical Education and Sports Science, National Institute of Education, Nanyang Technological University, Singapore, Singapore; ^2^Leiden Institute of Advanced Computer Science, Faculty of Mathematics and Natural Sciences, Leiden University, Leiden, Netherlands; ^3^School of Physical Education, Sport and Exercise Sciences, Division of Sciences, University of Otago, Dunedin, New Zealand; ^4^Physical Education and Sports Teacher Academy, Academy of Singapore Teachers, Ministry of Education, Singapore, Singapore

**Keywords:** nonlinear pedagogy, physical education, transfer of learning, traditional sporting games, territorial game

## Abstract

**Introduction:**

Nonlinear Pedagogy (NP), underpinned by Ecological Dynamics, provides a suitable pedagogical approach for practitioners (e.g., Physical Educators, coaches) to encourage exploratory learning that is learner-centered even in Traditional Sporting Games (TSG) that could be represented by invasion or territorial-like games. NP involves the manipulation of constraints which form boundaries for interacting components to self-organize, facilitating the emergence of goal-directed behaviours. Key pedagogical principles relating to representativeness, manipulation of constraints, awareness of focus of attention instructions, task simplification and the functional role of noise can encourage exploratory learning that helps develop 21st century competencies. This is in contrast to a Linear Pedagogy (LP) approach that is more teacher-centered and focuses on repetition in practices to promote movement form consistency in enhancing the acquisition of movement skills. Little is known about the effectivity of NP in the learning and transfer of invasion games. The aims of this study were to: (a) determine the impact of NP on the teaching and learning of an invasion game in the Physical Education (PE) context; (b) examine the transferability of game skills to other games in the same game category (i.e., floorball as a territorial game in this study).

**Methods:**

224 (between 12 to 13 years old) students underwent a 10-week intervention program to learn to play an invasion game (football) with either a NP or LP approach (i.e., repetitive and prescriptive drills).

**Results:**

Performance outcome data were measured during Pre, Post, Retention, Transfer test 1 (larger playing area) and Transfer test 2 (floorball). Significant improvements in several performance outcome and game play measures in football was observed for the NP condition. Fewer improvements in the same measures were found for the LP condition.

**Discussion:**

Evidence for transfer of learning for NP was not as strong as anticipated although there was still some potential for encouraging transfer of learning. The key findings from this study further challenge the “one-size fits all” philosophy in the teaching of PE. Both LP and NP approaches would have a role to play in supporting teaching and learning which could be context dependent.

## Introduction

A Nonlinear Pedagogy (NP) approach, based on Ecological Dynamics, has been advocated to provide practitioners with key pedagogical principles to support teaching and learning in the PE context (Chow, 2013; Komar et al., 2019; Renshaw and Chow, 2019; Button et al., 2020; Roberts et al., 2020). NP focuses on the inherent complexity involved in motor learning by viewing the individual learner, the task, the environment, and the teacher as key components of a complex interacting system (Chow et al., 2021). These multiple constraints operate on different time scales and interact among one another such that behaviour emerges due to these interacting constraints (Balagué et al., 2019). Specifically, NP is a pedagogical methodology providing principles that govern teaching effectiveness and efficacy in the learning process (Correia et al., 2019; Chow et al., 2022). Pertinent strategies on how to assess performance, how to structure practices, and how best to deliver instructions and provide feedback are particularly relevant (Ovens et al., 2013).

Learners should be given the opportunities to acquire individualized movement solutions based on the learning and performing context (Komar et al., 2019). The interaction of performer, environment and task constraints acknowledges the dynamic influences that each has on learning movement skills (Button et al., 2020). Balagué et al. (2019) further highlighted how task and task constraints, distributed between the performer and the environment, are emergent properties of the environment system. Encapsulating these constraints, practitioners can develop design principles that incorporate representativeness (i.e., *designing practices to mimic performance contexts that encourages the movement behaviours inherent in these contexts*), *manipulation of constraints* (i.e., *changing or accounting for performer, environment and especially task constraints*), *attentional focus* (i.e., *being sensitive to the impact of informational constraints that direct attention to movement form or movement outcome*), *functional variability* (i.e., *infusing practice variability to encourage exploratory behaviour to search for diverse functional solutions*), and the maintenance of pertinent information-movement couplings through *task simplification* (i.e., *reducing the complexity of the task without disrupting the relationship between perception and action for the movement such as increasing the size of object to be kicked or enlarging the playing area to reduce spatial and temporal challenges*; Chow, 2013). Importantly, these design principles, can help practitioners to promote skill transfer across a range of game-related concepts, including invasion of space, which are common to many traditional sporting games.

A growing body of empirical research examines the efficacy of using NP in physical education and sport coaching settings. For example, Lee et al. (2014), provided key insights on how NP can be effective in teaching a modified net-barrier game in a Singapore school setting. Primary school students with no tennis background underwent a learning intervention over several weeks that was taught with either a NP or Linear Pedagogy (LP) approach (i.e., a pedagogical approach with an emphasis on drill and technique to achieve a consistent optimal movement form). Changes in performance were observed at three levels: (1) the individual; (2) in a game setting; (3) in a class setting. Findings showed that the students in the NP condition were just as effective as the LP condition even though there were less explicit prescriptive instructions on movement form in the former condition. These findings provide evidence of *degenerate* behaviours amongst the NP-taught group where many different movement pathways could be recruited to achieve the same outcome (see Seifert et al., 2016).

Miller et al. (2016) also cited the use of NP ideas in enacting a game-centered approach intervention program to determine its effectiveness on improving physical activity and physical education outcomes. In an intervention program that involved 6 × 60 min PE lessons focused on the teaching of Fundamental Movement Skills (FMS) to children, it was found that there were improvements in throwing, catching, decision making and support during game play and in physical activity level during lessons. In another study by Miller et al. (2017), junior netball players were exposed to greater amounts of competition relevant activity, and hence better representativeness in the practices, that reportedly accounted for enhancements to decision making and support skills in game play. For both Miller et al. (2016) and Miller et al. (2017) studies, it was not clear exactly how the design principles in NP were incorporated nor how the interventions supported the control mechanisms for movement behaviours. It should also be noted that a game-centered approach is not specifically a NP approach (see Renshaw et al., 2016). In another study, Pizarro et al. (2019) examined the effects of a NP training program (over 12 training sessions) in the technical-tactical behaviour of youth futsal players. It was found that the players improved to some extent on their passing and dribbling as well as potential for greater ball possession but not in shooting at post intervention. However, this intervention was not undertaken within a PE setting with a small sample size (*N* = 8).

Importantly, there were no specific measurements of transferability of movement skills to other game contexts for the afore-mentioned studies. The issue of transferability is crucial as it highlights the adaptability of movement skills acquired in one context to another similar context. This is akin to learning a game skill in one category of game (e.g., territorial or invasion game) and how those skills may be transferable to another in the same category (e.g., from football to floorball). Such transferability would be important as PE time and resources in schools is typically limited and transferability of such learning across games of the same category could lead to more efficient learning of generic motor skills (Rudd et al., 2020).

The aims of this study were to: (i) determine the impact of NP on teaching and learning of an invasion game in the PE context; and, (ii) examine the transferability of game skills to other games in the same game category. It is predicted that NP would be more effective than LP in the PE context for a series of performance-based measures derived from the football (invasion game) lessons. Specifically, we predict that the NP lessons will develop learner’s abilities to play football by increasing the number of successful passes they make, more varied types of passes, longer duration that they remain in possession of the ball and higher number of goals scored. It is also predicted that NP would be more effective in supporting transfer of learning from one game to another within the same game category (i.e., from football to floorball).

## Methodology

### Participants

A quasi-experimental design was used in which four teachers were selected and their respective classes that were assigned to them were recruited for this study. In total, eight classes of Secondary one level students (*n* = 224) of between 12 to 13 years old at two schools were involved. The two schools were similar in size and social-economic profiles except that one school was a singe gender school (all boys) and the other was a mixed gender school. Classes recruited were then randomly assigned to a NP condition (*n* = 120) and a LP (*n* = 104) condition.

The same teachers (*n* = 4), with at least 3 years of teaching experience, taught both the NP and LP condition classes to control for the potential impact of teacher effectiveness on the two conditions. The teachers were well-prepared to teach a variety of sports and games using different pedagogical approaches (e.g., Games Concept Approach, Direct Instruction, Guided Discovery and a LP approach that focuses on achieving an expected movement form with an emphasis on drills) but had no prior experience using a NP approach but was subsequently prepared by the research team to teach with an NP approach as part of this study (please refer to information on intervention). The students were not informed if they would be in the NP or LP condition. Written parental consent and participant assent was obtained prior to the start of any data collection. Human ethics approval was granted by the University Institutional Review Board.

### Task

Students learnt game skills from an invasion game (football) within a PE context. All students involved in the study underwent a pre-test, intervention (10 weeks), post-test, retention and two transfer tests sessions. All tests were conducted within the student’s class groups during scheduled physical education lessons. Please refer to Table 1 for a visualization of the sequence of test and intervention sessions.

**Table 1:** Intervention schedule and program overview.

Week	Intervention Lesson	Games-related concept	Skills
1	-	Pre-test (over two sessions)	4v4 football game 4v4 floorball game
1	-	Pre-test (over two sessions)	4v4 football game 4v4 floorball game
2	1	Keeping possession of the ball	Passing, receiving and keeping possession
2	2	Keeping possession of the ball	Passing, receiving, keeping possession and shielding
3	3	Using space to invade	Ball control
3	4	Using space to invade	Moving and turning with the ball
4	5	Creating space to invade	Forward play
4	6	Creating space to invade	Switch play
5	7	Attacking the goal	Beating a player
5	8	Attacking the goal	Shooting
6	9	Stopping the invasion	Tackling
6	10	Stopping the invasion	Marking 1v1 defence
7	11	Regaining possession of the ball	Intercepting
7	12	Denying scoring opportunity	Getting goal-side
8	13	Denying scoring opportunity	Delaying
8	14	Denying space to invade	Defending as a team
9	15	Small-sided games	Revision and collation of skills taught
9	16	Small-sided games	Revision and collation of skills taught
10	-	Post test	4v4 football game
10	-	Post test	4v4 football game
11	-	Retention and Transfer test (1)	Retention and Transfer test (1)-football
11	-	Retention and Transfer test (1)	Retention and Transfer test (1)-football
12	-	Transfer test (2)	Transfer test (2)-Floorball
12	-	Transfer test (2)	Transfer test (2)-Floorball

**Figure 1:**
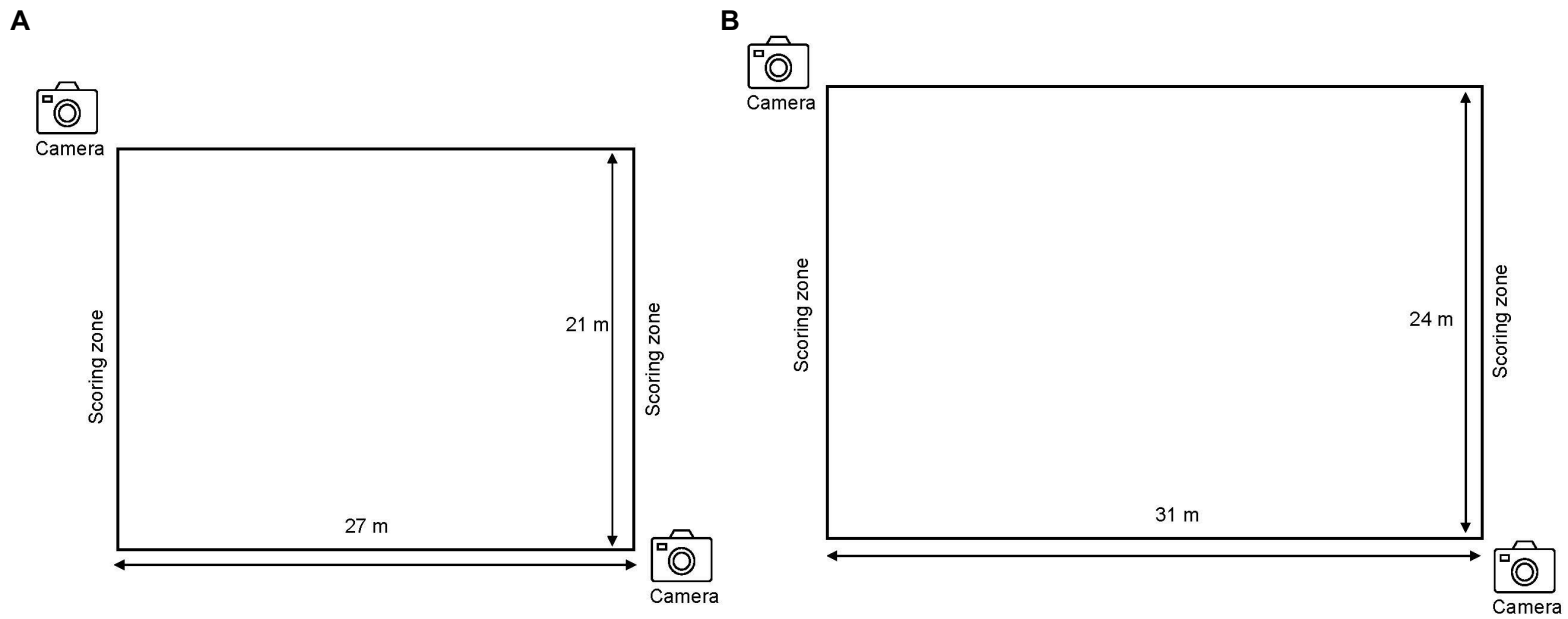
Illustration of the set-up for **(A)** pre-test, posttest and retention test sessions and **(B)** transfer test 1.

During the pre-test, post-test and retention test, students were required to play a small-sided 4v4 game of football with scoring zones at the ends of each length of the field with no goalkeeper. In order to score a goal, students had to move with the ball to the scoring zone or receive a pass from a teammate whilst in the scoring zone. The dimension of play was 21 m in width and 27 m in length. For the transfer test 1, students were tested on a novel context (larger playing field) in relation to the same game that they were taught. The dimension of play was 24 m in width and 31 m in length. The purpose of the transfer test 1 was to provide a platform to examine if students were able to adapt to a different playing area compared to the one that they were familiar with in terms of pitch dimension. In transfer test 2, students were tested using another invasion game (4v4 floorball) to determine transferability of learning (from football to floorball). The dimension of play was 21 m in width and 27 m in length. Floorball is an invasion game that is included in the Singapore PE syllabus and like football, floorball is played by two competing teams. The game is akin to a field hockey game where players manipulate a plastic floorball stick (similar to a field hockey stick) with a plastic ball (similar in size to a tennis ball) as they attempt to score a goal (similar in size to ice-hockey goal). See Figure 1 for an illustration of the set-up for the test sessions.

Throughout the different test sessions, students were kept to the same teams when possible and each team played against the same opposition. However, in instances where students could not attend the test sessions, in order to maintain consistency between sessions, it was ensured that no more than two students could be replaced per game. Students were replaced by another student of the same gender from the same class.

### Intervention

Students in the NP condition were presented with lessons that were underpinned by NP design principles. For example, NP learning activities incorporated many representative designs (e.g., small-sided games that provides relevant perceptual information), use of exploratory cues (e.g., analogies to encourage exploration of different movement solutions), infusing variability in practices (e.g., using difference ball sizes and playing dimensions in terms of space), a focus on simplifying task (e.g., modified rules and activities to encourage success). LP lessons focused on repetition of practices to acquire consistency in the expected movement solutions (e.g., kicking a ball based on prescriptive instructions on the form) and using standard sized equipment and playing areas to ensure practice specificity of the expected movement solutions. Examples of LP and NP lesson plans for the first week can be found in Supplementary Table 1. All intervention lessons were conducted during the students’ regular PE class times, which occurred during two 1-h slots every week.

Prior to the intervention lessons being delivered, the physical education teachers received 1-h weekly professional development sessions with the research team to ensure that the lessons on invasion games were delivered according to the lesson plans set out for the respective pedagogical conditions. Details of weekly lesson objectives can be found in Table 1. In addition, the lessons conducted by each teacher were supported by a research assistant in terms of administering and video-recording the lessons taught. A 2-h workshop on key pedagogical principles for both NP and LP was also delivered by the first author for the four teachers prior to data collection.

Evaluation of the intervention fidelity was determined by an external reviewer, independent to the study but familiar with PE pedagogy (including NP and LP), to ensure that the instructions undertaken by the teachers were in line with the intervention lesson plans. Two PE lessons per teacher per condition (e.g., 4 lessons from each teacher) were randomly selected to be observed and rated against a series of descriptors to confirm that the teachers did indeed abide to the planned lessons for both NP and LP conditions. Coding of the observed lessons by the research assistant (fourth author) showed a 78.6 and 82.1% lesson agreement for the NP and LP lessons, respectively.

### Data analysis

#### Performance outcome (football)

All test sessions (pre, post, retention, transfer test 1 and transfer test 2) were recorded using digital video cameras placed at two locations.

The research assistant coded the game performance outcomes. Performance outcome definitions and game coding details are outlined in Table 2. The entire game period was split into ‘runs’ which is defined as a period where the ball is in play for a minimum of 3 s. Performance outcome variables (e.g., passes, possession time and number of goals etc.) were then coded for each run. Table 3 provides coding details for the number of types of passes. Twenty percent of all test session videos (10% of LP and 10% of NP groups) were recoded and rated again (see Miller et al., 2016) and a percent agreement reliability test (number of agreements/number of agreements + number of disagreements) was used to assess the intra-rater reliability (Blomqvist et al., 2005). Reliability of game coding was found to be at 85% for NP and 86% for LP.

**Table 2:** Performance outcome definitions and game coding details.

Action	Definition
Pass	Successful pass When a teammate kicks the ball and the ball is received by his/her teammate Unsuccessful pass Opponent intercepts, gets possession of the ball or the ball goes out of play
Consecutive passes	Successful consecutive pass Team maintains possession of the ball when two or more continuous passes occur between teammates Unsuccessful consecutive pass Opponent intercepts, gets possession of the ball or the ball goes out of play
Goal	Successful goal Ball is brought into the scoring zone or the ball stopped within the scoring zone by a teammate after receiving a pass Unsuccessful goal Opponent intercepts, gets possession of the ball or the ball goes out of play
Possession time	Possession time starts from the moment the team is in possession of the ball to the time when the ball was intercepted, interrupted by the other team or is kicked or caught out of bounds
Turnover	Point in which the possession switches from one team to another
Runs	Period in which the ball is in play for more than 3 s

**Table 3:** Coding for number of types of passes.

Type	Type definition	Contact	Flight/Grounded	Outcome
Push pass	Pushing action on the ball	Inside foot	Flight	Into space
Hard pass (pass with more power)	Pass with power	Instep/Lace portion	Flight (low)	Direct
Chip pass	Ball kicked from underneath	Toe	Grounded	Direct to chest
Toe flick	Light touch/flicking action with the toe	Ankle		Direct to knee
Toe pass	Pass with toe (like a toe poke)	Sole		
Back pass	Passing the ball backwards	Outside foot		
Sweep pass	Sweeping action towards the ball rather than a simple push	Back of foot		
Drag pass	Contact/Dragging the ball between the bottom of the foot and the ground	Knee		
Off ground passes	Any passes that involve contact with the ball above ground			

Before inferential statistics were performed, preliminary assumption testing was conducted on all variables. Specifically, checks were conducted for multivariate normality using the Shapiro–Wilk test of normality, univariate and multivariate outliers using the Mahalanobis distance, homogeneity of variance–covariance matrices through the Box’s M Test of Equality of Covariance Matrices and multicollinearity. In instances where the assumption of normality was violated, Pillai’s Trace was used to evaluate the multivariate significance of the principal effects. Outliers found through the use of Mahalanobis distance were removed from the analysis. In the case where homogeneity of covariance was violated, Pillai’s Trace was used instead of Wilk’s Lambda to evaluate the multivariate significance of the principal effects and the interactions.

Multivariate analyses (MANOVA) 2 (Group: NP and LP) X 4 (Session: Pre-test, Post-test, Retention test, Transfer test 1) were conducted to determine differences within groups and between pedagogical conditions for performance outcomes (e.g., mean successful passes, number of consecutive passes, number of types of passes, total possession time and number of goals). Separate MANOVAs were conducted for performance outcome, game play behaviours during runs. It was insightful to explore the possible differences in game dynamics that could surface during a run as it is an important feature of invasion games. A mixed factorial ANOVA was conducted for the variable of turnover counts.

### Transfer of learning from football to floorball

A 2 (Group: NP and LP) X 2 (Pre-test and Transfer test 2) MANOVA was used to determine the differences within and between groups for performance outcomes (e.g., mean successful passes, total number of goals and total possession time).

All statistical analyses were conducted using IBM SPSS Statistics 26.0 at significance level of *p* < 0.05 and effect size was calculated using partial eta squared η*_p_*^2^. In instances when the MANOVA detected statistically significant differences, univariate ANOVAs with Bonferroni post-hoc test was conducted on the simple effects of interest.

## Results

### Performance outcome

Significant improvements in performance outcome in football (e.g., mean successful passes and number of types of passes) were observed for the NP groups. See Table 4 for a summary of results for performance outcome across sessions.

**Table 4:** Summary of results across sessions.

Performance outcome		
Type	NP	LP
Mean successful passes	More mean successful passes during post-test and retention test compared to pre-test	No changes across sessions
Number of types of passes	Higher number of types of passes during post-test, retention test and transfer test compared to pre-test	No changes across sessions
Total possession time	Higher possession time from post-test compared to pre-test	Higher possession time from post-test, retention test and transfer test 1 compared to pre-test
Number of consecutive passes	No changes across sessions	No changes across sessions
Total number of goals	No changes across sessions	No changes across sessions
Turnover counts	Higher turnover count during post-test compared to pre-test	Higher turnover during transfer test 1 compared to pre-test
Transfer of learning from football to floorball		
Type	NP	LP
Mean successful passes	More mean successful passes at transfer test 2 compared to pre-test	No changes across sessions
Total possession time	Higher possession time at transfer test 2 compared to pre-test	No changes across sessions
Total number of goals	No changes across sessions	Higher total number of goals at transfer test 2 compared to pre-test

There were significant main effects of Group [Pillai’s Trace = 0.061, *F*(3, 190) = 4.10, *p* = 0.008] and Session [Pillai’s Trace = 0.183, *F*(9, 576) = 4.15, *p* = <0.001] on the combined dependent variables of mean successful passes, number of consecutive passes, number of types of passes, total possession time and total number of goals. There was no significant interaction effect between Group x Session [Pillai’s Trace = 0.062, *F*(9, 576) = 1.35, *p* = 0.210]. Between Sessions, follow-up univariate test found a significant difference for mean successful passes, *F*(3, 192) = 6.15, *p* = 0.001, η*_p_*^2^ = 0.088. The NP group experienced more successful passes during post (13.21 ± 5.28, *p* = 0.003) and retention test (13.00 ± 5.25, *p* = 0.006) as compared to pre-test (8.50 ± 4.77), however there were no significant changes for the LP group across test sessions.

**Figure 2:**
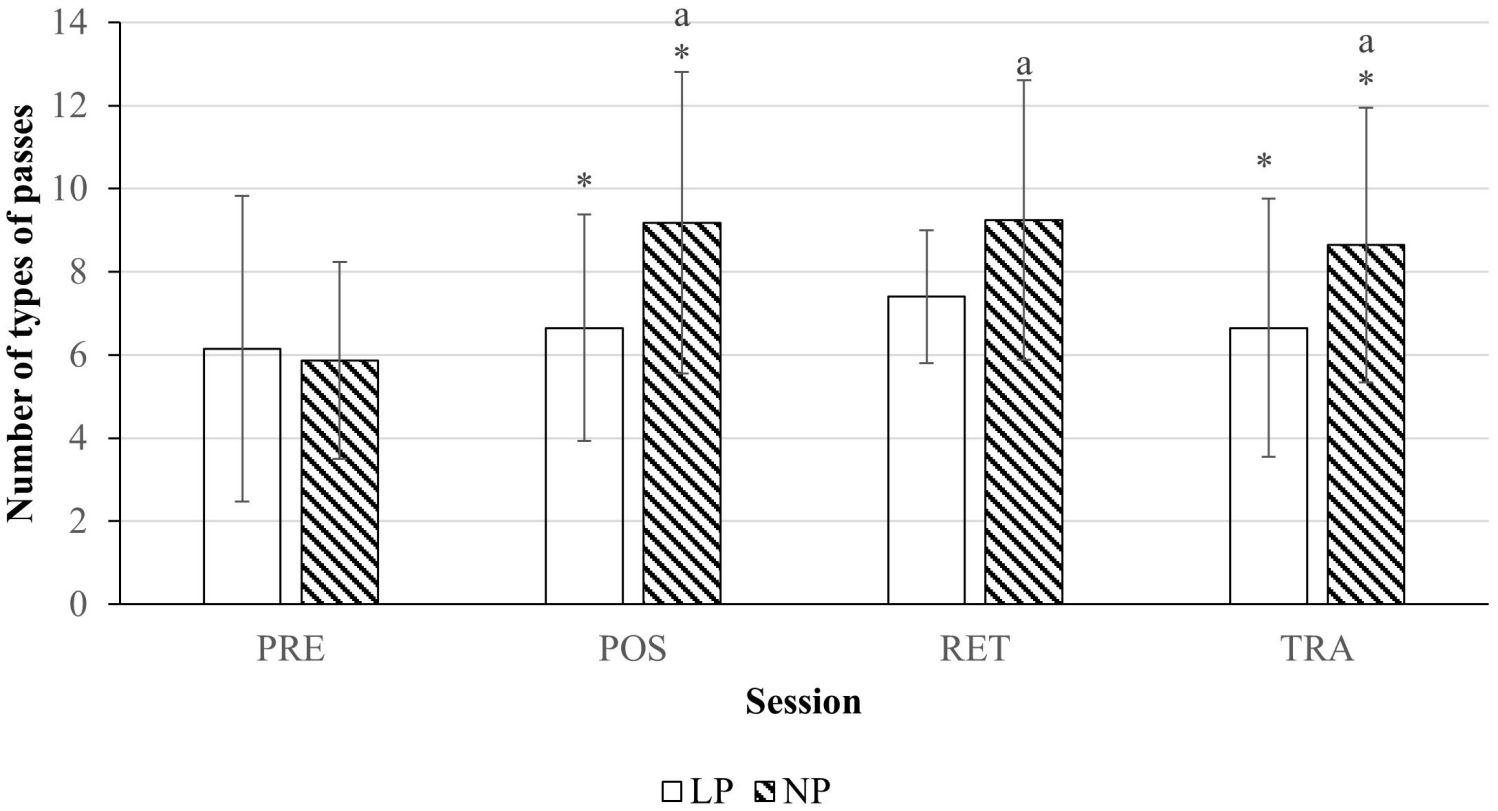
Number of types of passes. (*) Indicates significant differences between groups (*p* < 0.05) and (a) indicates significant differences in comparison to the pre-test (*p* < 0.05). The x-axis shows pre-test (PRE), post-test (POS), retention test (RET) and transfer test (TRA) sessions. Transparent bar represents LP condition and shaded bar represents NP condition.

Separate univariate ANOVAs revealed significant main effects of Group [*F*(1, 184) =11.438, *p* = 0.001, η*_p_*^2^ = 0.059] and Session [*F*(3, 184) = 5.134, *p* = 0.002, η*_p_*^2^ = 0.077] on the number of types of passes (see Figure 2). Specifically, only NP groups experienced a higher number of types of passes during post-test (9.18 ± 3.62; *p* < 0.001), retention test (9.25 ± 3.36; *p* < 0.001) and transfer test 1(8.64 ± 3.30; *p* = 0.005) from pre-test (5.86 ± 2.37). No significant changes were found for LP across sessions. Between Groups, NP had more types of passes as compared to LP during post-test (*p* = 0.005) and at transfer test 1 (*p* = 0.028).

A significant univariate main effect of Session was also obtained for total possession time, *F*(3, 192) = 11.47, *p* < 0.001, η*_p_*^2^ = 0.152. NP group showed higher possession time from post-test (87693.21 ± 27410.44, *p* = 0.010) to pre-test (63113.57 ± 27341.91). LP group experienced significantly higher possession time from post (90642.27 ± 32696.68, *p* = 0.001), retention (97014.09 ± 31644.32, p < 0.001) and transfer test 1 (89235.45 ± 33310.86, *p* = 0.002) to pre-test (57183.64 ± 24105.14). There was no main effect of Group, *F*(1, 192) = 1.68, *p* = 0.197, η*_p_*^2^ = 0.009.

Univariate ANOVAs for number of consecutive passes and total number of goals showed that there was no significant main effects for Session and Group.

For Turnover counts (i.e., change in possession between teams) there were significant main effects of Session [*F*(3, 100) = 6.68, *p* < 0.001] and Group [*F*(1, 100) = 6.99, *p* = 0.010], however there was no significant interaction effect. *Post hoc* test revealed that between Sessions, NP groups has a significantly higher turnover count (*p* = 0.048) during post-test (26.13 ± 11.85) as compared to pre-test (17.00 ± 7.54), whereas this significant difference was found (*p* = 0.001) for LP group at transfer test 1 (25.75 ± 11.89) from pre-test (11.08 ± 7.23).

### Transfer of learning from football to floorball—Performance outcome

Significant improvements in performance outcome during the transfer of learning to floorball (e.g., mean successful passes and total possession time) were observed for the NP groups. There was a significant main effect Session [Pillai’s Trace = 0.173, *F*(3, 78) = 5.42, *p* = 0.002] on the combined dependent variables of number of passes, number of goals and possession time. There was no significant main effect of Group and no significant interaction effect.

**Figure 3:**
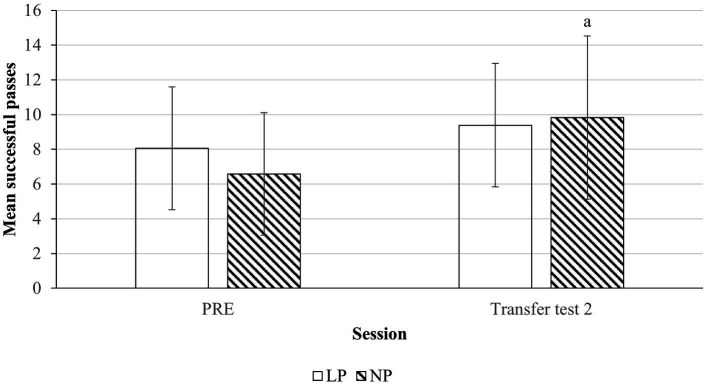
Mean successful passes for floorball between pre and transfer test 2 sessions. (a) Indicates significant differences between Pre and Transfer test 2 (*p* < 0.05). The x-axis shows pre-test (PRE), transfer test 2 sessions. Transparent bar represents LP condition and shaded bar represents NP conditions.

Univariate tests revealed that for mean successful passes (see Figure 3), there was a main effect of Session, *F*(1, 80) = 7.08, *p* = 0.009, η*_p_*^2^ = 0.081. *Post hoc* analysis showed that the mean successful passes for NP group was significantly higher at transfer test 2 (9.83 ± 4.70, *p* = 0.005) from pre-test (6.58 ± 3.53). LP group did not experience significant changes between Sessions (*p* = 0.309). There was no significant interaction effect, indicating that at transfer test 2, mean number of passes was not different between the two groups (*p* > 0.05).

With regards to the total number of goals, a main effect was found for Sessions, *F*(1, 80) = 9.11, *p* = 0.003, η*_p_*^2^ = 0.102. Between Sessions, the LP group had higher total number of goals during Transfer test 2 (2.44 ± 2.09, *p* = 0.011) from pre-test (0.89 ± 1.18). NP group did not experience any statistical change across Sessions (*p* > 0.05). There was no Group and interaction effect as well (*p* > 0.05).

A main effect of Session was also found for total possession time, *F*(1, 80) = 9.12, *p* = 0.003, η*_p_*^2^ = 0.102. *Post hoc* analysis showed that total possession time was significantly different between sessions only for NP (*p* = 0.003) from pre (80074.25 ± 27699.09) to transfer test 2 (104020.92 ± 28546.13). Similarly, there was no Group and interaction effect (*p* > 0.05).

## Discussion

The aims of this study were to: (i) determine the impact of NP on teaching and learning of an invasion game in the PE context; and, (ii) examine the transferability of game skills to other games in the same game category (i.e., football to floorball). Findings from this study have added new knowledge to the impact of NP on the teaching, learning and transfer of invasion games. Significant improvements in several performance outcome and game play measures in football was observed for the NP condition. Fewer improvements in the same measures were found for the LP condition. Specifically, NP demonstrated more successful passes and number of types of passes between sessions. LP demonstrated higher possession time from post, retention and transfer test 1 compared to pre-test, while there was only a significant difference for NP between pre and post-test sessions. NP also had higher turnover count during post-test as compared to pre-test whereas LP showed difference at transfer test 1 to pre-test. With regards to transfer of learning to a floorball game, NP had higher mean successful passes but LP had higher total number of goals at transfer test 2 from pre-test, respectively. Total possession time was significantly higher only for NP from transfer test 2 to pre-test. Nevertheless, there was less clarity on the benefits for either NP or LP conditions for transfer from football to floorball.

One important insight from these results was confirmation that key pedagogical principles for NP can be effective in PE settings. A larger range of functional movement behaviours (e.g., greater number of different types of passes) observed in NP classes further affirmed that an exploratory pedagogical approach was as good as a prescriptive pedagogical approach (LP) to teaching invasion games with reference to enhancing performance outcomes. This is especially insightful as it suggests that a NP approach to teaching game skills allows students to explore their perceptual motor landscape more effectively to acquire a greater variety of movement behaviours, harnessing the degenerate features of the human movement system more actively (Button et al., 2020). This is in contrast to a LP approach where the students are more likely to adhere to the prescribed movement cues and limit exploration of other possible movement solutions. In terms of practical implications, the NP approach could promote a broader range of passing solutions in actual game performance contexts. Specifically, the inclusion of representative small-sided games in NP approach could support students to acquire higher potential of functional diversity such that they can better adapt to different game and environmental constraints (Hristovski and Balagúe, 2020). A greater repertoire of passing solutions will intuitively provide greater opportunities to execute more functional play outcomes as compared to a context where students are only using a limited array of passing options as prescribed in the LP lessons. This could explain the higher number of successful passes found in the NP condition. Such a variety of passing options could indeed lead to greater effectiveness to achieve the task goal present in game situations. This in turn can be manifested in greater observed adaptability on the part of the learner which is a key in supporting transfer of learning. Through NP, teachers can manipulate task constraints (e.g., design of practices and use of appropriate informational constraints) to encourage learners to search, explore and exploit different movement solutions (Roberts et al., 2019; Button et al., 2020). These exploratory behaviours are important because they help learners to become attuned to information that matches environmental properties and the learner’s own capabilities to move (Hacques et al., 2020). Undoubtedly, from a specificity of practice perspective, one would expect that multiple solutions should be practiced if flexibility in using multiple solutions is the desired outcome of learning (Ranganathan et al., 2020).

It was also noted that while both NP and LP conditions could cope equally well when they had to play on a larger pitch size (transfer test 1), a greater advantage resided in the NP classes. The transfer to a novel context in terms of different pitch dimensions do offer opportunities for students to adapt their learning. Skill adaptation is a key learning outcome in PE since lesson time for NP, which focuses on exploratory learning, is likely to be a premium within a school curriculum (Chow, 2013). Moving forward, other possibilities in terms of transfer test within the same game (i.e., football in this case) may be undertaken to examine how NP and LP conditions could possibly elicit different responses. For example, the addition of goals or changes in number of players would be alternatives in examining adaptability within the game of football. This is particularly pertinent when consideration is to be given to how the impact of an NP approach based on ecological dynamics can be assessed. Indeed, skill transfer and learning should be seen as an adaptive process to support skill and talent development (Seifert et al., 2018).

While the performance outcomes were promising for a NP approach to teaching football, the results on transfer across different games (from football to floorball) in the same game category did not emerge as strongly as anticipated for NP. Note that NP demonstrated more effectiveness for *number of successful passes*, and *total possession time*, but LP had the advantage for *total number of goals*. Nevertheless, one limitation of the work is the use of the current variables to examine impact of NP or LP approach to invasion games. It should be recognised that especially in invasion games, the behaviour of teams and players are closely connected to how fellow teammates and opponents play. There is inherent limitation to discrete performance outcome variables in such a context where cooperation and opposition co-exist simultaneously in space and time.

It is also possible that perhaps the transfer test of floorball could be too different from the game of football in terms of game play dynamics and the effect of transfer did not manifest itself as significantly. It may signal that the gap between the intrinsic dynamics of the students (i.e., the inherent movement repertoire of the students) is probably too big with reference to what is needed for the transfer task, which is in this case the floorball task. Specifically, the task dynamics of the floorball task could differ significantly from the task dynamics of the football task (i.e., the need to manipulate a stick in floorball could be a transfer scenario that is distinct from football even though both are territorial games). Thus, while the intention is to consider the possibility of transfer, it is critical to examine the dynamics of the task. Transfer may be potentially challenging even though the games could be in the same game category when the task dynamics differ significantly. This lends itself to the discussion on general or specific transfer of learning and the role of specifying and non-specifying information. Specific transfer refers to a context where the practice closely relates to how the movement would be performed while general transfer refers to practices where the movement is less related to how it would actually be executed in a performance context but provides some approximation of its execution. In ecological dynamics, specifying information is functionally relevant for regulating human behaviours and is deemed to be more useful for specific transfer contexts while non-specifying information is more meaningful for general transfer contexts (Chow et al., 2020). The information available in the football context (e.g., ball in relation to use of feet for control) could be more specifying for a transfer to a game that closely resembles football (e.g., indoor football like futsal) rather than floorball in this context. Maybe larger time scales (i.e., months of training) or more tests could also be needed to evaluate the transfer of football to floorball as well.

Nevertheless, the key findings from this study further challenge the “one-size fits all” philosophy held by many PE teachers (see Lee et al., 2014). Practitioners should see themselves as co-designers of practices and as facilitators of learning (Correia et al., 2019; Roberts et al., 2019). Importantly, practitioners should consider alternate assessment rubrics rather than those focused solely on a ‘correct’ movement form expected of all students when designing practices and lessons. While NP may be perceived to be more time-consuming to enact and perhaps less ‘control’ in how students would explore movement solutions, the benefits come in the form of the learning experiences (Chow, 2013).

In this study, NP was observed to be as good as LP or better in relation to performance outcomes. This is encouraging as one might expect that experiences of the two approaches would differ markedly where NP is more learner-centered in encouraging the learner to solve movement problems themselves and self-organize under constraint. Nevertheless, the use of LP could still be relevant as in reality, teachers are probably more likely to use some form of hybrid approach that incorporates elements of both NP and LP approaches. The difference could be the extent to which NP or LP actually featured in some of these practice sessions (see Supplementary Table 1). The polarization of using either NP and LP in this current study is intentionally structured and future work could involve a mixed pedagogy that has a blend of both NP and LP to examine its effects on learning in TSG. Ideas pertaining to LP where stabilization of an optimal pattern and repetition may work well for some situations cannot be discounted (i.e., for safety reasons, or if the learner is too nervous to explore). But more often than not, striving for exploration and adaptability could work even more effectively.

Moving ahead, key 21^st^ century competencies such as creativity, cooperative-competitive intelligence, and team-work should be assessed in the future (Hristovski and Balagúe, 2020). There could also be better ways to examine if and how learning occurs within the context of team games in TSG especially in relation to adaptation. Measuring performance outcome is one of many possible indicators of learning but understanding changes in team dynamics in small-sided games would also be insightful (e.g., how teams adapt to one another with NP, LP or even a mixed approach). Undoubtedly, future work on examining NP can also have further implication on Teacher Education and Professional Development programs pertaining to PE. Pedagogical principles pertaining to NP can be relevant for revising and enhancing PE curriculum in schools (Chow et al., 2021). Notably, there is potential to use NP to encourage teaching and learning to be student-centered and focusing on optimizing individual movement competencies (Chow et al., 2021). Practitioners could incorporate key pedagogical principles of NP in the delivery of content and teaching within school-based PE settings for TSG to help develop greater creativity and involvement among students. Translation of the findings and implications on practice can also be examined in future studies.

## Data availability statement

The datasets presented in this article are not readily available because only anonymized data may be available on request. Requests to access the datasets should be directed to jiayi.chow@nie.edu.sg.

## Ethics statement

The studies involving human participants were reviewed and approved by Nanyang Technological University Institutional Review Board (IRB-2015-02-029). Written informed consent to participate in this study was provided by the participants’ legal guardian/next of kin.

## Author contributions

JC, CB, and BT conceptualized and designed the study. CC collected the data. LM and CC performed the data analysis. JC and CC wrote most of the paper with LM and CB providing valuable input. All authors contributed to the article and approved the submitted version.

## Funding

This paper refers to data from the research project “Nonlinear Pedagogy and its relevance for the new PE curriculum” (OER 21/14 CJY), funded by the Education Research Funding Program, National Institute of Education (NIE), Nanyang Technological University, Singapore. The views expressed in this paper are the authors and do not necessarily represent the views of NIE.

## Conflict of interest

The authors declare that the research was conducted in the absence of any commercial or financial relationships that could be construed as a potential conflict of interest.

## Publisher’s note

All claims expressed in this article are solely those of the authors and do not necessarily represent those of their affiliated organizations, or those of the publisher, the editors and the reviewers. Any product that may be evaluated in this article, or claim that may be made by its manufacturer, is not guaranteed or endorsed by the publisher.
