# Supplementary material for: The effect of nonlinear pedagogy on the acquisition of game skills in a territorial game
Source: Front Psychol. 2023 Feb 6;14:1077065. doi: 10.3389/fpsyg.2023.1077065 (PMC9940013; doi:10.3389/fpsyg.2023.1077065)
Supplement: Supplementary file 1 [file Table_1.docx]

Supplementary Table 1. *Week 1 Lesson Plans – Linear Pedagogy and Nonlinear Pedagogy*

| **Lesson time: 60 min** | **Linear pedagogy – Lesson 1**  **Number of students: 35 to 40** | **Objectives:**  Keeping possession of the ball  (Passing, receiving and keeping possession) |  |  |  |
| --- | --- | --- | --- | --- | --- |
| **Time** | **Activity** | **Teaching cues/ Instructions** | **Organisation/ Equipment** | **Pedagogical Principles** | **Mechanisms** |
| 10 mins | **3v1 Possession Game**   - Warm-up activity - 3 students to keep ball with 1 student as the ‘IT’   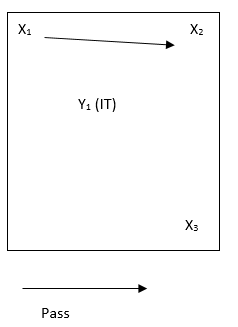 | **Cues and Instructions**   - No specific rules given on how to pass or receive - Any touches by the 3 players who have possession of the ball - The three players are required to stay at any of the four corners in the grid - Keep activity within the grid | - 11 x 13m (split the quarter of the field into 8 equal parts) | - Game-like practice | - Contextual Interference |
| 10 mins | **Pair Work**   - A pair to a ball - a) Pass and receive (Stationary) - b) Pass and receive (Receiver to move to new passer) | **Cues and Instructions**   - *Passing:* - Flex your ankle and bend your kicking leg - Use the inside of the shoe to contact ball - Non-kicking foot beside the ball - *Receiving:* - Receive the ball with the flat surface of the shoe - Keep your eye on the ball | - Quarter of a field | - Attention focus (Internal and prescriptive) - Focus on repetitive drills to develop consistency in kicking action | - Conscious and explicit knowledge - Task decomposition |
| 10 mins | **4 in a grid**   - a) Passes around the grid - b) Passes across the grid   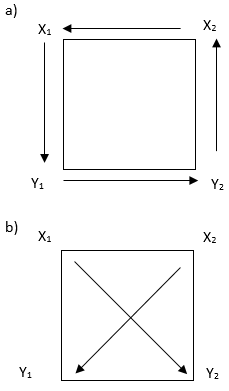 | **Cues and Instructions**   - Use the teaching cues and instructions shared in the previous activity - Begin with any touches; Proceed to 3 touches and finally to 2 touches - Begin with one ball and then move on to using two balls in the same grid - Try to keep the rally going for as long as possible | - 11 x 13m (split the quarter of the field into 8 equal parts) | - Attention focus (Internal and prescriptive) - Focus on repetitive drills to develop consistency in passing and reception of passes - Increase contextual interference with the inclusion diagonal passes and an additional ball in the practice | - Conscious and explicit knowledge - Repetitive practice - Contextual Interference |
| 15 mins | **4v4 Game**   - 4v4 invasion game - Emphasis on pass and receive   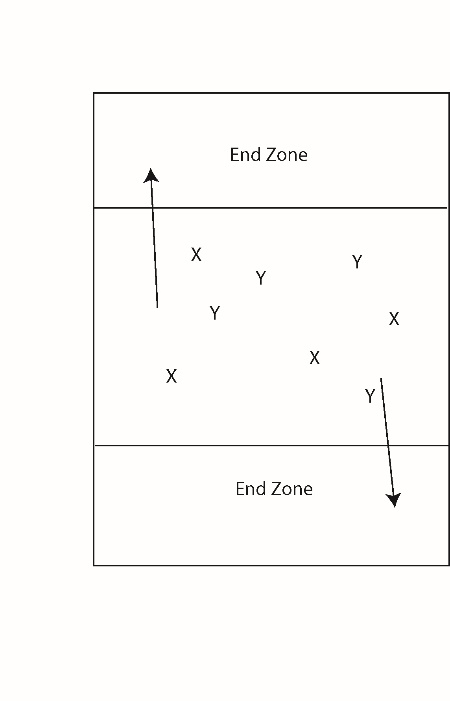 | **Cues and Instructions**   - Use previous cues taught for passing and receiving during earlier practice - Scoring by receiving a pass from a team mate in the end zone - Begin with any touches; Proceed to 3 touches if possible - Progress from big to smaller sized grids | - 4 grids - Grid size: - Small: 11 x 13m - Big: 11 x 26m | - Game-like practice - Linear progression of skill complexity | - Contextual Interference - Building of movement representation in a progressive approach |
| 5mins | **Summary**   - Gather in a group | **Cues and Instructions**   - Recap points on passing and receiving | - As per usual arrangement for all summary recap |  |  |

| **Lesson time: 60 min** | **Nonlinear pedagogy – Lesson 1**  **Number of students: 35 to 40** | **Objectives:**  Keeping possession of the ball  (Passing, receiving and keeping possession) |  |  |  |
| --- | --- | --- | --- | --- | --- |
| **Time** | **Activity** | **Teaching cues/ Instructions** | **Organisation/ Equipment** | **Pedagogical Principles** | **Mechanisms** |
| 10 mins | **3v1 Possession Game**   - Warm-up activity and introductory game - 3 students to keep ball with 1 student as the ‘IT’   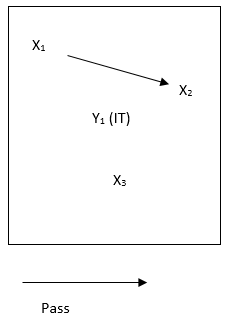 | **Cues and Instructions**   - No specific rules given on how to pass or receive - Any touches by the 3 players who have possession of the ball - Keep activity within the grid | - 8 grids - Grid size: 11 x 13m | - Representativeness | - Building representativeness for transfer |
| 10 mins | **Pair Work**   - A pair to a ball - a) Pass and receive (Stationary) - b) Pass and receive (Receiver to move to new passer) | **Cues and Instructions**   - *Passing:* - Ball to team mate’s feet - Allow pass to be easily controlled - Keep ball on the ground - *Receiving:* - Feet like a cushion/ pillow - Ball out to the side | - Quarter of a field - Use different size and type of balls - Use softer and bigger size balls | - Attentional focus (External and the use of analogies) - Task simplification - Variability in practice - Constraints manipulation | - Sub-conscious and implicit acquisition of movement - Strengthening of Information-movement coupling - Exploration and degeneracy - Manipulation of control parameters |
| 10 mins | **3v1 Possession Game**   - a) Back to introductory game - 3 students to keep ball with 1 student as the ‘IT’ - b) Progress to moving ball/possession from one end of the grid to the other end   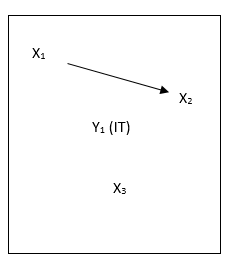 | **Cues and Instructions**   - As per earlier warm up game - Use the teaching cues and instructions shared in the previous activity - Begin with any touches; Proceed to 3 touches and finally to 2 touches | - 8 grids - 11 x 13m | - Representativeness - Variability in practice - Constraints manipulation | - Building representativeness - Exploration and degeneracy - Manipulation of control parameters |
| 15 mins | **4v4 Game**   - 4v4 invasion game - Emphasis on pass and receive   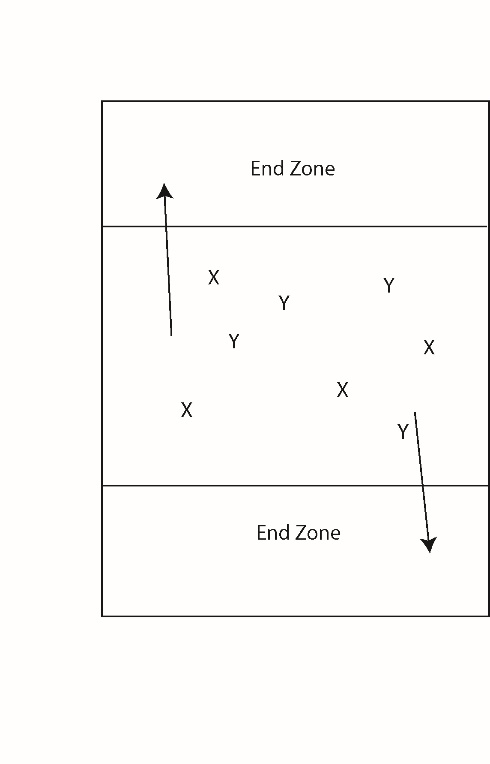 | **Cues and Instructions**   - Building on previous activity (3v1) game - Scoring by receiving a pass from a team mate in the end zone - Begin with any touches; Proceed to 3 touches if possible - Use different size grids (random rotation of teams to different sized grids) - Using different size balls | 4 grids   - Grid size: - Small: 13 x 16.5m - Big: 11 x 26m | - Representativeness - Variability in practice - Constraints manipulation | - Building representativeness - Exploration and degeneracy - Manipulation of control parameters |
| 5mins | **Summary**   - Gather in a group | **Cues and Instructions**   - Recap points on passing and receiving | - As per usual arrangement for all summary recap |  |  |
